# Adherence to Auto-CPAP and Age: A Stable Condition? Comment on Barroso et al. Influence of Age on Adherence to Auto-CPAP: Experience from a Sleep Center in Portugal. *Adv. Respir. Med.* 2022, *90*, 143–147

**DOI:** 10.3390/arm90040045

**Published:** 2022-08-18

**Authors:** Ahmet Cemal Pazarlı, Antonio M. Esquinas

**Affiliations:** 1 Department of Pulmonary Diseases, Faculty of Medicine, Gaziosmanpasa University, Tokat 60200, Turkey; 2 Intensive Care Unit, Hospital Morales Meseguer, 30008 Murcia, Spain

We have read with great interest the recent paper published by Barroso et al. entitled “Influence of Age on Adherence to Auto-CPAP: Experience from a Sleep Center in Portugal”. This interesting study revealed that patients over 65 years of age have adequate adherence to CPAP treatment [1]. However, although this study is in line with other studies published, we feel that there are some points that should be considered for correct interpretation of the results.

The authors have not included some relevant information about changes in the Epworth Sleepiness scale (ESS) as well as factors that are associated with the prognosis in terms of sleep-related symptoms and the quality of life [2]. In addition, they have ignored data regarding patients aged over 80 years. From a neurological point of view, some authors have found no differences in the use of CPAP and have not demonstrated any effect on any neurocognitive tests (including anxiety and depression) [3]. Another important aspect that would be nice to know is whether the authors consider that the patients need educational, supportive, and behavioral interventions to maintain adherence to CPAP in the long term [4]. Moreover, providing information regarding the correlation between the socioeconomic conditions of patients and adherence to CPAP could have been useful. [5] In addition, the authors could have compared their results with other geographical areas of Portugal as there may be a geographical factor, and it would be interesting to see how the geographic factor affects the results [6]. Finally, the authors stated in the limitations of their study that they do not know which different APAP models, as well as interfaces, could be adapted. In addition to the limitations they indicated, we think it would be appropriate for the authors to state that OSA phenotypes are also important in PAP adherence [7].

We believe that future studies on adherence to CPAP in patients >65 years of age could establish other non-respiratory factors regarding their capacity for adherence stability and accessibility to this type of CPAP treatment.
